# Monocyte/macrophage inflammatory response pathways to combat *Francisella* infection: possible therapeutic targets?

**DOI:** 10.3389/fcimb.2014.00018

**Published:** 2014-02-21

**Authors:** Devyn D. Gillette, Susheela Tridandapani, Jonathan P. Butchar

**Affiliations:** Department of Internal Medicine, Wexner Medical Center, The Ohio State UniversityColumbus, OH, USA

**Keywords:** *Francisella*, monocytes, macrophages, signaling, host response

## Abstract

*Francisella tularensis* can bypass and suppress host immune responses, even to the point of manipulating immune cell phenotypes and intercellular inflammatory networks. Strengthening these responses such that immune cells more readily identify and destroy the bacteria is likely to become a viable (and perhaps necessary) strategy for combating infections with *Francisella*, especially given the likelihood of antibiotic resistance in the foreseeable future. Monocytes and macrophages offer a niche wherein *Francisella* can invade and replicate, resulting in substantially higher bacterial load that can overcome the host. As such, understanding their responses to *Francisella* may uncover potential avenues of therapy that could promote a lowering of bacterial burden and clearance of infection. These response pathways include Toll-like Receptor 2 (TLR2), the caspase-1 inflammasome, Interferons, NADPH oxidase, Phosphatidylinositide 3-kinase (PI3K), and the Ras pathway. In this review we summarize the literature pertaining to the roles of these pathways during *Francisella* infection, with an emphasis on monocyte/macrophage responses. The therapeutic targeting of one or more such pathways may ultimately become a valuable tool for the treatment of tularemia, and several possibilities are discussed.

## Introduction

*Francisella tularensis* is the Gram-negative causative agent of tularemia (Sjostedt, 2007). *F. tularensis* has been classified into distinct subspecies, including *F. tularensis* subsp. *tularensis* (*F. tularensis*; Type A), *F. tularensis* subsp. *holarctica* (*F. holarctica*; Type B), and *F. tularensis* subsp. *novicida (F. novicida)*, which may actually be a separate species (Johansson et al., 2010). *Francisella* is especially recognized for its low infectious dose and ability to cause severe illness and death, which justifies its categorization as a Category A select agent by the USA Centers for Disease Control and Prevention (CDC) (Sjostedt, 2007). Of note, the most life-threatening forms of tularemia are particularly associated with Type A infections regardless of host species (Mohapatra et al., 2013). Although known to infect a range of host organisms and cell types (Rick and Wu, 2007; Hall et al., 2008), *F. tularensis* has evolved to successfully infect human monocytes/macrophages where the bacteria escape the phagosome, replicate within the cytosol and then move on to other cells as the infected cells die (Clemens and Horwitz, 2007; Elkins et al., 2007; Jones et al., 2012; Celli and Zahrt, 2013). *In vivo*, macrophages appear to be the preferred host cell for *Francisella* (Sjostedt, 2003; Elkins et al., 2007).

One critical characteristic of *F. tularensis* is its ability to attenuate host inflammatory responses. Indeed, early studies in humans showed that *Franicsella*-infected individuals exhibited diminished cytokine responses to endotoxin (Greisman et al., 1963). In the murine system *F. tularensis* infection does not lead to a classic pro-inflammatory cytokine response, and this results in insufficient numbers of immune cells recruited to infection sites (Bosio et al., 2007). Further, murine studies have corroborated the findings of Greisman et al., (1963), who found that challenge with lipopolysaccharide (LPS) after infection did not lead to the production of pro-inflammatory cytokines such as Tumor Necrosis Factor α (TNFα) in mouse cell lines nor *in vivo* (Telepnev et al., 2003, 2005; Bosio, 2011). Similar findings have also been observed in *F. tularensis*-infected murine bone-marrow and alveolar macrophages following administration of the synthetic triacylated lipopeptide Pam_3_CSK_4_ (Crane et al., 2013).

Circulating monocytes constitute lower than 10% of blood immune cells, yet serve a critical role as primary responders to infection (Moser and Loetscher, 2001; Leavy, 2012). As mentioned above they are also prime targets of *Francisella* infection. Along with this, a higher percentage of monocytes are infected by *F. tularensis* than either *F. holarctica* or *F. novicida* during the course of infection (Hall et al., 2008). The focus of this brief review is on some of the critical intracellular response pathways involved with *Francisella* infection. The role of each pathway during *F. tularensis* infection is summarized, with an emphasis on monocyte/macrophage responses. Following this is a short discussion of potential non-antibiotic means of combating *Francisella* by modulating these response pathways.

## *Francisella* and toll-like receptors

Host immune cells have evolved to contain an array of receptors which are vital for the detection of invading microbes and foreign materials. These surface- and endosomally-expressed sensors, termed pattern recognition receptors (PRR), can recognize highly conserved bacterial, viral, and fungal motifs (Brown et al., 2011). Toll-Like Receptors (TLR) are key PRR that are expressed by a variety of cells including monocytes and macrophages. *Francisella* directly interacts with the host cell through TLR2, a surface receptor that recognizes surface lipopeptides and peptidoglycan. In fact, TLR2^−/−^ mice infected with *F. tularensis* Live Vaccine Strain (LVS) display markedly lower TNFα and Interleukin 6 (IL-6) production, decreased Nuclear factor kappa-light-chain-enhancer of activated B cells (NF-κB) activation, and showed overall lower survival when compared to wild-type mice (Katz et al., 2006; Malik et al., 2006). Specific lipopeptides that can induce TLR2 signaling, particularly the triacylated 17-kDa membrane lipoprotein FTT0901/TUL4/LpnA (Sandstrom et al., 1987; Sjostedt et al., 1989, 1991) and the membrane lipoprotein FTT1103/FipB (*Francisella* infectivity potentiator protein B) (Qin and Mann, 2006; Qin et al., 2011), are present on *Francisella*'s surface (Thakran et al., 2008).

Modifications in TLR2 expression levels are associated with *Francisella* infections, and these can vary depending on the subspecies. For example, we have shown that *F. novicida* significantly increased TLR2 transcript after infection in primary monocytes while *F. tularensis Schu S4* decreased it (Butchar et al., 2008). In addition to altering receptor expression, both *F. tularensis* and *F. novicida* can downregulate the molecule Cluster of differentiation 14 (CD14) (Butchar et al., 2008). This is an important co-receptor for both TLR2 and TLR4. It is required by the host cell in order to generate a potent pro-inflammatory cytokine response to *F. tularensis* LVS, although it is not adequate for increasing survival *in vivo* (Chase and Bosio, 2010). In like manner, recent reports highlight the role of the downstream adapter Myeloid differentiation primary response 88 (MyD88), as mice lacking this molecule died rapidly when challenged with *F. tularensis* LVS (Collazo et al., 2006).

The importance of TLR4 in responding to LPS has long been recognized (Chow et al., 1999; Qureshi et al., 1999). *Francisella*, however, expresses an atypical LPS that does not strongly induce TLR4 (Duenas et al., 2006). This is attributed to the lack of two acetyl groups in its LPS, making it incapable of inducing a strong TLR4 response (Phillips et al., 2004). TLR signaling appears to be very effectively subverted by *Francisella*, and this may position TLR as well as downstream pathway members as prime candidates for targeted therapy (discussed in a later section).

## Inflammasome responses

Cystolic sensing mechanisms such as the multi-protein inflammasome play a prominent role in recognizing intracellular pathogens, including *Francisella*. This depends upon *Francisella*'*s* escape from the phagolysosome (Mariathasan et al., 2005; Gavrilin et al., 2006). Inflammasomes regulate caspase activation through proteolytic cleavage, leading to Interleukins 1β and 18 (IL-1β and IL-18) processing. Cleavage of procaspase-1 to caspase-1 requires TLR2, as TLR2^−/−^ mouse macrophages showed little caspase-1 24 h after infection with LVS (Dotson et al., 2013). Expectedly, caspases have been implicated in the regulation of *Francisella* infections. For example, mice lacking caspase-1 displayed higher bacterial numbers in organs following infection with *F. novicida* (Mariathasan et al., 2005; Jones et al., 2010).

*Francisella*'s escape from the phagosome triggers Absent in Melanoma 2 (AIM2) inflammasome activation, as a subset of *F. novicida* have been shown to lyse within the cytosol and release AIM2-activating double-stranded deoxyribonucleic acid (dsDNA) (Fernandes-Alnemri et al., 2010; Jones et al., 2010; Rathinam et al., 2010). The importance of AIM2 in *Francisella* infection was clearly demonstrated by a study showing that AIM2^−/−^ mice had increased organ bacterial burden and lower survival rates than wild-type following infection with *F. novicida* (Fernandes-Alnemri et al., 2010). In humans however, it has been shown that the NALP3 inflammasome was the primary driver of IL-1β production, with AIM2 contributing as well (Atianand et al., 2011).

The influence of *Francisella* on inflammasome activity appears to be subspecies-dependent. For example, recent data suggest that *F. novicida* does not inhibit inflammasome activation (Broz and Monack, 2011). In contrast, it has been shown that LVS delays inflammasome activation and cell death, an activity that requires the OmpA-like *Francisella* protein FTL_0325. *In vivo*, mice infected with LVS harboring a mutation in FTL_0325 showed significantly higher IL-1β by the first day after infection while mice infected with control LVS showed higher IL-1β at day 3. Importantly, mice infected with the LVS mutant that permitted earlier inflammasome activation showed a significantly lower bacterial load at day 3 (Dotson et al., 2013).

Virulent *Francisella* manipulates inflammasome responses by stimulating the activation of apoptosis-promoting caspase-3 rather than caspase-1 (Wickstrum et al., 2009; Bosio, 2011). Tissues from Type A *F. tularensis* infected mice expressed increased cleaved caspase-3, in contrast to the tissue responses of *F. tularensis* LVS- and *F. novicida*-infected mouse macrophages. In these cells, an increased caspase-1 dependent/caspase-3 independent inflammatory cytokine production was more evident (Wickstrum et al., 2009).

## Type I and II interferons

Interferons (IFNs) are host-produced proteins with an inherent role in pathogen clearance during infection. These Type I interferons induce signal transduction molecules, upregulate major histocompatibility complex (MHC) molecules and promote proliferation of T cells (Welsh et al., 2012). Importantly, they have been shown to be critical for inflammasome activation in response to *Francisella* (Henry et al., 2007). While usually associated with viral infections, interferons are also seen with *Francisella* infection. For example, *F. novicida* is able to induce a variety of Type I IFN-associated genes in mouse bone marrow-derived macrophages (BMM) (Henry et al., 2007), and Schu S4 upregulated IFNβ in human peripheral blood monocytes (Butchar et al., 2008). However, it has been shown that *Francisella* suppresses Type I interferon signaling. For example, the virulent *F. tularensis* strain Schu S4 inhibited the ability of dendritic cells to produce IFNα and IFNβ (Chase et al., 2009). Infection of human monocytes with Schu S4 led to downregulation of IFNα receptors 1 and 2 (Butchar et al., 2008). As such, it appears as though the more virulent form of *Francisella* uses more than one means to combat Type I IFN signaling.

It has also been shown that infection of human primary monocytes with *F. tularensis* and *F. holarctica* not only leads to downregulation of Type I interferon pathway components but also Type II (Butchar et al., 2008; Cremer et al., 2011). IFNγ, a cytokine produced primarily by natural killer (NK) and T cells, regulates the immunological response to effectively clear pathogens. IFNγ can lower bacterial number following infection with LVS (Anthony et al., 1989; Polsinelli et al., 1994), and can reduce the intra-macrophage growth of LVS in a dose-dependent manner (Anthony et al., 1992). Later reports demonstrated that macrophages treated with IFNγ were more efficient in clearing *Francisella* via an increased ability to perform phagosome-lysosomal fusion (Santic et al., 2005). In human monocytes, both *F. tularensis* and *F. novicida* increased IFNγ ligand expression but decreased IFNγ Receptor 1 (Butchar et al., 2008). In conjunction, it has been shown in both human and murine monocytic cell lines that *F. novicida* induces Suppressor of Cytokine Signaling 3 (SOCS3) expression, suppresses Signal Transducer and Activator of Transcription 1 (STAT1) phosphorylation, and suppresses both Interferon gamma-induced protein 10 (IP-10) and Inducible Nitric Oxide Synthase (iNOS) production (Parsa et al., 2008a).

Because of the *Francisella*-mediated dampening of both Type I and Type II interferon signaling, there is a possibility that pharmaceutical delivery of interferons may help combat infection. Intron A (Spiegel, 1985), Rebif (Mantia et al., 2013), and Actimmune (Todd and Goa, 1992) are clinically approved drugs that deliver interferons alpha, beta, and gamma to the patient, respectively. They have been utilized for the management of Multiple Sclerosis, Chronic Granulomatous Disease (CGD), and Hepatitis B infection, but there is a possibility that one or more may aid against at least some forms of tularemia.

Another *Francisella* family member, *F. philomiragia*, is an opportunistic pathogen found with immunocompromised individuals. In particular, it is associated with the abovementioned CGD, which can lead to fatal septicemia (Seger et al., 1982; Mailman and Schmidt, 2005). Interestingly, both *F. tularensis* and *F. philomiragia* have been associated with chronic granulomas and necrotizing abscesses (Schmid et al., 1983; Nylund et al., 2006). Of particular importance, *F. philomiragia* has between a 70–85% homology to *F. tularensis* (Whipp et al., 2003), suggesting that both pathogens may to some degree respond to IFN treatment. However, Melillo et al., (2010) showed that IFNγ did not improve the ability of human macrophages to combat Schu S4. Further testing, perhaps with the use of monocytes, or using IFNγ plus other agents, may uncover a positive role of IFNγ against virulent *Francisella*.

## The role of NADPH

Target host cells of *Francisella* can respond to infection with the production of reactive oxygen species (ROS) and reactive nitrogen species (RNS) (Lindgren et al., 2005). Here we will focus on ROS, which are generated following the assembly of nicotinamide adenine dinucleotide phosphate-oxidase (NADPH oxidase) and are a crucial innate defense mechanism. Not surprisingly, however, *Francisella* has devised an array of techniques to inhibit ROS. These include techniques focused on hindering NADPH component assembly, obstructing ROS production from assembled NADPH oxidases and neutralizing generated ROS (Bosio, 2011; Jones et al., 2012). *Francisella*, including both virulent and less virulent strains, reduces ROS production in neutrophils and macrophages. The acid phosphatase AcpA has been shown to be important for inhibiting reactive oxygen species production in both macrophages and neutrophils (Mohapatra et al., 2010). Another report showed that in neutrophils, virulent *F. tularensis* both with and without an AcpA mutation, suppressed the production of superoxide anions from the NADPH oxidase complex (McCaffrey et al., 2010).

The live vaccine strain of *F. tularensis* was able to persist within neutrophils by avoiding acquisition of gp91/p22 plasma membrane and p47/p67 cytosolic NADPH subunits (McCaffrey and Allen, 2006). This supported the growth of *F. tularensis* LVS by hindering NADPH assembly. The importance of altering NADPH complexes is not unique to *Francisella*, as multiple bacteria including *Helicobacter pylori* and *Salmonella typhimuriu*m have been shown to alter NADPH oxidase assembly in cells (Gallois et al., 2001; Allen et al., 2005). If *Francisella* does encounter ROS, catalases and super oxide dismutases (SOD) enzymes are necessary for survival, as ΔSOD *F. tularensis* LVS have increased susceptibility to IFNγ-induced death (Melillo et al., 2009). Indeed, it has been shown that antioxidants produced by *Francisella* Schu S4 can dampen macrophage inflammatory responses (Melillo et al., 2010).

## The PI3K/Akt pathway

Phosphatidylinositol 3^′^-kinase (PI3K) leads to activation of Akt, also known as protein kinase B (PKB/Akt) [please see (Hers et al., 2011; Hemmings and Restuccia, 2012) for brief reviews on the PI3K/Akt pathway, and (Cremer et al., 2011) for a short review within the context of *Francisella*]. The cellular processes mediated by PI3K include phagocytosis (Araki et al., 1996), autophagy (Petiot et al., 2000), cytokine production (Parsa et al., 2006), and oxidative burst (Chen et al., 2003; Hoyal et al., 2003). Hence, manipulation of PI3K may be advantageous for pathogens. For example, macrophages from PI3K-deficient mice show impaired nitric oxide production and increased predisposition to *Chlamydia pneumoniae* infection (Sakai et al., 2006). Stimulation of the PI3K/Akt pathway has downstream positive effects on NF-κB activation and host response (Rajaram et al., 2006). It has also been reported that PI3K and Akt are crucial in the production of RANTES (“regulated on activation, normal T cell expressed and secreted”), IL-6, and IL-12 following *F. novicida* infections (Parsa et al., 2006; Rajaram et al., 2006). *F. tularensis* Schu S4 but not *F. novicida* leads to downregulation of the regulatory p85 subunit of PI3K, as well as Akt itself (Butchar et al., 2008). Conversely, mice expressing constitutively active Akt (MyrAkt) did not succumb to *F. novicida* infections to the same extent as wild-type mice (Rajaram et al., 2006). Initiation of these pathways favors the host largely through the activation of NF-κB, which promotes survival, cytokine production, and phagosomal maturation (Telepnev et al., 2005; Parsa et al., 2006). However, it has also been reported that wortmannin, by blocking Akt activation and mitogen-activated protein kinase phosphatase 1 (MKP1) upregulation, could enhance Mitogen-activated Protein Kinase 1 (MAPK1) and phosphorylation of the p38 MAPK, as well as cytokine release in murine BMM following infection with LVS (Medina et al., 2010). It was also shown that Complement component 3 (C3) opsonization of Schu S4 led to phosphorylation of Akt in human monocyte-derived macrophages (MDM) and that this led to an upregulation of the Erk inhibitor MKP-1 (Dai et al., 2013). Further experiments may be needed to tease out the role(s) of Akt during *Francisella* infection, but the differences seen are likely due to differences in complement, in bacterial subspecies and/or cell type. The results of Dai et al., however, point to the C3 pathway as a putative therapeutic target.

Downstream inhibitors of PI3K have also been shown to be involved with dampening responses following *Francisella* infection. Deletion of a key phosphatase, Phosphatidylinositol-3,4,5-trisphosphate 5-phosphatase 1 (SHIP1) has been shown to permit greater cytokine production following infection of primary murine macrophages with *F. novicida* (Parsa et al., 2006). In addition, Phosphatase and tensin homolog (PTEN) is upregulated following *Francisella* Schu S4 infection of human MDM (Melillo et al., 2010). Both phosphatases serve to dampen PI3K activity, resulting in a lessening of responses such as cytokine production. SHIP1 has also been shown to attenuate Ras activity by binding Shc (Damen et al., 1996). MicroRNAs (miRs) are post-transcriptional regulators of gene expression and *Francisella* has developed methods to use microRNAs to its advantage (Cremer et al., 2009; Eulalio et al., 2012). Specifically, miR-155, which targets the 3^′^ UTR of SHIP1, is induced by *F. novicida* but not Schu S4, resulting in higher levels of SHIP1 with Schu S4 (Cremer et al., 2009).

## The Ras pathway

*Francisella* also modulates the Ras-Raf-MAPK kinase-MAPK signaling pathway during infection (Al Khodor and Abu, 2010; Asare and Abu Kwaik, 2010). It has been shown that intracellular *F. novicida* triggers Ras activation within 15 min in human MDM. This occurs through Son of Sevenless 2 (SOS2)/Growth factor receptor-bound protein 2 (GRB2)/Protein kinase C α (PKCα) and Protein kinase C β 1 (PKCβ 1), which are essential for bacterial proliferation (Al Khodor and Abu, 2010). Along with proliferation, the Ras pathway has been linked to cell death associated with *Francisella* infection. Inhibition of MAPK1 phosphorylation prevented LVS-induced apoptosis in the J774.2 mouse macrophage cell line (Hrstka et al., 2005). Uptake of *F. novicida* also depends on MAPK1, via activation of Spleen tyrosine kinase (Syk) (Parsa et al., 2008b). Ras activation upon infection is not specific to *F. novicida*, as *Listeria monocytogenes* and *Helicobacter pylori* promote Ras activation during infection (Keates et al., 2001; Mansell et al., 2001).

In contrast to observations with *F. novicida*, it has been shown that C3-opsonized Schu S4 dampens activation of MAPK1, p38 MAPK, and NF-κB, along with cytokine production in human monocyte-derived macrophages (Dai et al., 2013). Cytokine responses to non-opsonized Schu S4 were stronger, although not as strong as the responses to *F. novicida*, and C3 opsonization did not alter the responses to *F. novicida* (Dai et al., 2013). These C3-mediated dampening effects appeared to be due to activation of the protein tyrosine kinase LYN (Dai et al., 2013). Hence, it appears as though there are differences in response to *F. novicida* and *F. tularensis* that suggest caution when making inferences from one to the other. Additional studies will be required in order to tease out the intricacies of virulent *Francisella* and Ras.

Effectively targeting the Ras pathway may provide a novel means of combating *F. tularensis*. Celecoxib, an FDA-approved cyclooxygenase 2 (COX-2) inhibitor normally administered as an anti-inflammatory agent, has recently been implicated in the upregulation of MAPK1 and/or p38 MAPK activity in head and neck squamous cell carcinoma cell lines, inhibiting their proliferation (Park et al., 2010). Importantly, a potent antimicrobial activity of celecoxib and a derivative has been reported, which appears specific against *Francisella*. Celecoxib and a pharmacologic derivative termed Compound 20, killed *F. novicida*, LVS, and Schu S4 in growth media. In addition, compound 20 inhibited the growth of *F. novicida* and Schu S4 in Raw 264.7 mouse macrophage cells (Chiu et al., 2009). Hence, celecoxib or related compounds may offer a dual effect against *Francisella*: promoting host cell responses and direct killing.

## Can we fight *Francisella* without antibiotics?

Research to date points to immunosuppression as a critical factor in the virulence of *Francisella*. This leads to the hypothesis that enhancing inflammatory responses would serve to combat infection. Although cytokines such as TNFα, IL-1β, and IFNγ are known to activate certain aspects of cellular immune responses and are known to be attenuated by *Francisella*, treatment with these or other such agents may not be sufficient to combat an antibiotic-resistant form of this pathogen. For example, even though IFNγ may promote phago-lysosomal fusion upon infection with the less virulent *F. novicida* (Santic et al., 2005), it does not appear to protect human MDM against Schu S4 (Melillo et al., 2010). Hence, although IFNγ may be important for combating *Francisella*, it in itself is not sufficient. Likewise, the specific therapeutic targeting of other pathways may not be sufficient to mount a successful immune response against *Francisella*. Another approach may be to stimulate the production and activation of monocytes through administration of a factor such as Granulocyte-macrophage colony-stimulating factor (GM-CSF). This factor acts on both monocytes and neutrophils, and has been tested extensively as an antitumor agent (Waller, 2007). Along with this, it can enhance the activity, including respiratory burst, of monocytes as has been shown *ex vivo* with septic patient monocytes (Williams et al., 1998). Although GM-CSF did not reduce intra-macrophage growth of LVS (Anthony et al., 1992), there is a possibility that it may show some efficacy *in vivo*.

Alternatively, it is likely that the simultaneous activation of multiple immune response pathways will be required. One potential non-antibiotic-based treatment that has already made its way into the cancer arena is the use of immunomodulatory agents. Indeed, this is being actively pursued as a potential treatment for sepsis as well as for several viral and bacterial infections (reviewed in Savva and Roger, 2013).

Immunomodulators have been studied and used for the treatment of cancer for well over 100 years. Tumors exert a strong immunosuppressive effect on host immune responses, even to the point where they co-opt immune cells for the production of factors that promote growth, survival, and angiogenesis (Becker et al., 2013; Kushner and Bautch, 2013). From this perspective, perhaps there are enough similarities between *Francisella* and tumor cells with regard to immunosuppression [e.g., both involve Transforming growth factor β (TGFβ) production (Bosio et al., 2007; Becker et al., 2013)] that these compounds would be effective in treating tularemia. In fact, it has recently been shown that administration of a TLR4 agonist conferred protection against *F. novicida* infection in mice (Lembo et al., 2008). It was later shown that a mix of DNA-liposome complexes plus *Francisella* membrane fractions could protect mice from *F. tularensis* infection (Ireland et al., 2010).

The first promising immunomodulator described in the literature was Coley's Toxin, produced by Coley, (1891). This was a mix of bacteria that typically resulted in fever and malaise after injection but oftentimes led to the reduction or elimination of the patients' tumors. Since then, research has uncovered mechanisms both by which host immune cells respond to such “toxins” and by which tumor cells act to suppress immune responses (Becker et al., 2013; Broz and Monack, 2013). A later bacterially-based therapy was bacillus Calmette-Guerin (BCG), used as a tuberculosis vaccine and subsequently approved for treating bladder cancer (Vacchelli et al., 2013). Synthetic agents were also being developed such as imiquimod (Chen et al., 1988) and resiquimod (Tomai et al., 1995). Imiquimod (brand name Aldara) was approved in 1997 for the treatment of genital warts and certain skin cancers. Although such compounds are developed on an ongoing basis, the common theme is that as TLR agonists, they possess the ability to activate multiple immune-response-related pathways simultaneously (Brown et al., 2011). This fuller spectrum of activation, in contrast to single-pathway treatment such as with Interferons, carries the potential to more effectively combat *Francisella* infection.

Monocytes express most TLR, although with low levels of TLR9 and virtually no TLR3 (Hornung et al., 2002), so it is likely that most immunomodulators will lead to their activation. Perhaps as importantly, activation of monocytes with these compounds can also indirectly elicit responses from neighboring cells. For example, the TLR7/8 agonist resiquimod promoted the production of IFNγ from Natural Killer (NK) cells *in vitro*, but only through monocyte-derived IL-12 during co-culture (Hart et al., 2005). Direct or indirect effects on other cells have been well-documented as well. For example, resiquimod has been shown to promote dendritic cell maturation and antigen presentation (Ahonen et al., 1999), and treatment of PBMC with the TLR8-selective agonist VTX-2337 led to enhanced dendritic cell maturation as well as more effective priming of CD8^+^ T cells (Stephenson et al., 2013). Hence, although monocytes respond strongly to such agonists, functional responses *in vivo* and in humans will result from the culmination of direct and indirect responses among multiple cell types. Although immunomodulators continue to be developed, it may be beneficial to begin testing the efficacy of agents that are currently approved for other uses in humans. For example, BCG and even perhaps the topically-applied Imiquimod could be tested in mouse models. There are numerous mouse models of infection that can be utilized (Conlan et al., 2011), so a plethora of possibilities exists.

Other than the well-characterized side effects associated with the use of immunomodulators such as fever, nausea and malaise (Witt et al., 1993; Goldstein et al., 1998; Pockros et al., 2007; Weigel et al., 2012), there are additional considerations. For example, as demonstrated by Ireland et al. with macrophages (Ireland et al., 2010), the timing of immunomodulator administration may be a critical factor in the efficacy of treatment. With *Francisella* in particular, the more virulent *F. tularensis* Schu S4 has been shown to alter the expression and function of immune response factors. For example, most TLR (as well as the MyD88 adaptor protein) in monocytes are downregulated following infection with *F. tularensis* (Butchar et al., 2008). Similarly, it has been shown that *F. tularensis* can block NF-κB activation, PKB/Akt phosphorylation and cytokine production in macrophages (Melillo et al., 2010). Hence, within the context of tularemia it would be hoped that enough monocytes/macrophages (and other cells) would come into contact with the agonist before encountering *Francisella*, such that a more effective immune response could be attained. Along with timing, it is important to consider that repeated dosages of immunomodulators may not be fully effective. Endotoxin tolerance following an initial stimulus can lead to hyporesponsiveness to subsequent stimuli (Greisman and Hornick, 1975; West and Heagy, 2002; Morris and Li, 2012). It has been shown in a mouse model of tumor immunotherapy that systemic administration of the TLR7/8 agonist resiquimod led to such hyporesponsiveness, which was overcome by altering the timing of repeated injections (Bourquin et al., 2011). Based on this, it seems probable that immunomodulators by themselves will not be fully effective against *Francisella*.

The use of therapeutic antibodies within the cancer field has been ongoing since 1997 with the advent of Rituximab, and several others are in use or testing for a variety of cancers. Much of their efficacy has been attributed to antibody-dependent cellular cytotoxicity (ADCC) (Sliwkowski et al., 1999; Clynes et al., 2000), which monocytes are capable of performing (Shaw et al., 1978). There have also been efforts to engineer these antibodies for better binding, and/or for drug delivery to target cells (Vincent and Zurini, 2012).

Antibodies within the context of *Francisella* have also been examined preclinically, with promising results. (Stenmark et al., 2003; Stenmark and Sjostedt, 2004). Of particular note, human serum from a person infected with *F. tularensis holarctica* was able to confer protection against *F. tularensis holarctica* in mice (Stenmark et al., 2003). Stenmark and Sjostedt went on to show that immune serum led to increases in both TNFα and IL-12 (Stenmark and Sjostedt, 2004), which had been previously shown to be important within the context of *Francisella* infection (Stenmark et al., 1999). From a practical perspective, it may not be feasible to isolate anti-*Francisella* antibodies from people who survived infection and use them to treat currently-infected patients. In addition, there are currently few commercially-available antibodies against virulent *F. tularensis*. However, DNA-based technology has made the production of monoclonal antibodies far less cumbersome so it is not unreasonable to predict that a battery of humanized antibodies could be available in the future. Furthermore, as with several antitumor antibodies, such new anti-*Francisella* antibodies may be engineered to enhance binding and/or immunogenicity. Due to the immunosuppressive nature of *F. tularensis*, however, it is possible that anti-*Francisella* antibodies alone will not be fully effective for all patients.

Perhaps a combination of immunomodulators and anti-*Francisella* antibodies should be explored, as it has been shown that the two together can lead to superadditive immune responses. For example, we found that treatment of human monocytes with the TLR7/8 agonist resiquimod led to synergistic increases in IgG-mediated TNFα production. Resiquimod also enhanced monocyte-mediated ADCC against a tumor cell line and synergistically improved the efficacy of antitumor antibody therapy *in vivo*. Interestingly, resiquimod modulated not only the function but also the expression of monocyte Fcγ receptors (FcγR), such that activating receptors were upregulated and the inhibitory FcγRIIb was downregulated (Butchar et al., 2010). Similarly, the TLR8-selective agonist VTX-2337 was shown to increase the effectiveness of NK cell-mediated ADCC (Lu et al., 2012). Although not all TLR agonists may modulate monocyte FcγR expression to equal extents, it is likely that at least one or more FDA-approved agents such as BCG could. Within the context of tularemia, this dual therapy might successfully combat the immunosuppressive effects of *F. tularensis* and direct the host immune cells specifically against this pathogen. It remains to be tested, however, whether such treatment can offset the *Francisella*-mediated suppression seen upon contact and phagocytosis. Lastly, from a treatment point of view, the synergistic effects of dual treatment might permit the use of lower dosages and thereby minimize untoward effects.

## Conclusion

*Francisella* has evolved methods to escape and suppress host cell immune responses. This might be counteracted via the use of immunomodulatory agents or antibodies, and the combination of both may lead to the best results. Further research may lead to the successful development and testing of such agents.

### Conflict of interest statement

The authors declare that the research was conducted in the absence of any commercial or financial relationships that could be construed as a potential conflict of interest.
